# Cranial hypertrophic pachymeningitis with myelodysplastic syndrome

**DOI:** 10.1016/j.heliyon.2024.e32973

**Published:** 2024-06-13

**Authors:** Shohei Kikuchi, Tomohiro Hayashi, Honoka Nitta, Yusuke Kamihara, Akinori Wada, Tomoki Minemura, Yoshimi Nabe, Jun Murakami, Yuji Nakatsuji, Tsutomu Sato

**Affiliations:** a Department of Hematology, Toyama University Hospital, Toyama, Japan; b Department of Neurology, Toyama University Hospital, Toyama, Japan; c Department of Clinical Laboratory and Blood Center, Toyama University Hospital, Toyama, Japan; d Division of Transfusion Medicine and Cell Therapy, Toyama University Hospital, Toyama, Japan

**Keywords:** Hypertrophic pachymeningitis, Myelodysplastic syndrome, Steroids therapy

## Abstract

Hypertrophic pachymeningitis (HP) is a rare inflammatory disease characterized by thickening of the dura mater. HP develops with several inflammatory diseases. Antineutrophil cytoplasmic antibody (ANCA)-associated vasculitis and IgG4 related disease are reported as 2 major causes. With hematologic diseases, only 3 cases have been reported. We report the case of myelodysplastic syndrome (MDS) developing HP. Our case provides a thought-provoking hypothesis regarding the potential relationship between MDS and HP.

## 1 Introduction

Hypertrophic pachymeningitis (HP) is a rare inflammatory disease characterized by thickening of the dura mater. Clinically, thickening of the dura mater causes intracranial hypertension resulting in severe headache. Pathologically, interstitial fibrosis and inflammatory cell infiltration consistent with inflammatory disease are observed. HP secondarily develops with a variety of inflammatory diseases such as autoimmune disease and infections. Antineutrophil cytoplasmic antibody (ANCA)-associated vasculitis and IgG4-positive multifocal fibrosclerosis-related systemic diseases (IgG4-RD) are reported as 2 major causes of HP [1]. With hematological disease, only 3 cases have been reported in association with HP; i.e., Myelodysplastic syndromes (MDS), aplastic anemia and monoclonal gammopathy of undetermined significance (MGUS) [[2], [3], [4]]. MDS are a heterogenous group of clonal myeloid neoplasms in hematopoietic stem cells characterized by dysplastic and ineffective hematopoiesis. The immune system is compromised in MDS patients, which can influence the disease's progression or lead to the development of various autoimmune diseases [5]. We report the case of MDS developing HP suggesting the potential relationship between MDS and HP via immune response.

## 2 Case report

**Fig. 1:**
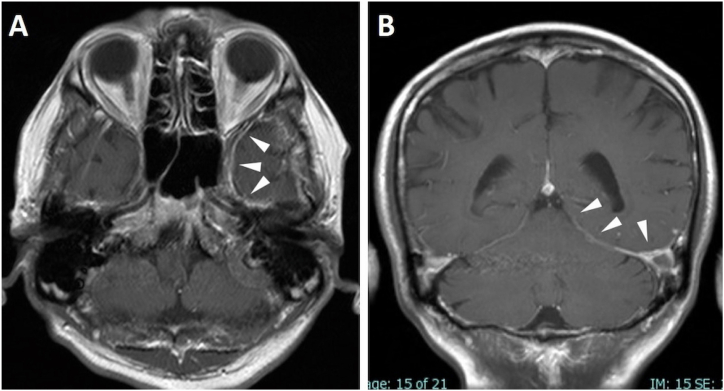
T1-weighted gadolinium-enhanced magnetic resonance imaging (MRI) of the brain at diagnosis. Enhancement and thickening of dura matter were observed predominantly on the left tentorium with horizontal (A) and coronal (B) view (arrows).

A 68-year-old male was found to have pancytopenia with 1770/μL of white blood cell count, 6.6 g/dl of hemoglobin concentration and 11.5 × 10^4^/μL of platelet count at a different hospital in April 2020 (Table 1). He had no particular comorbidities other than cholelithiasis and vitreous disease. Bone marrow examination revealed dysplasia with 3 lineages and 4.5 % myeloblasts (Table 2). Chromosomal analysis using the G-banding method showed complex abnormalities, including chromosome 7 deletion (Table 3). The patient was diagnosed with MDS with multilineage dysplasia according to the WHO criteria and was classified as high risk according to the revised international prognostic scoring system. He was referred to our clinic, and a careful review of the patient's history revealed that he had complained of headache at the same time as the onset of MDS. Gadolinium-enhanced brain MRI revealed dura matter thickening with an enhanced effect on the left middle cranial fossa, leading to the diagnosis of cranial HP (Fig. 1A&B). C-reactive protein and ESR were slightly elevated. Serological examinations for underlying autoimmune diseases such as ANA, MPO-ANCA, and PR3-ANCA were all negative (Table 1). Infectious diseases such as syphilis or tuberculosis, which are known etiologies of HP, were also excluded by blood examinations. A cerebrospinal fluid examination showed no abnormal findings with normal pressure, normal biochemistry test results, no cellular infiltration, and normal cytology. Although the etiology of HP remained unclear, because of severe and acutely intensified headache requiring opioid use, systemic steroid therapy with pulse-dosed methylprednisolone was administered before azacitidine therapy. After methylprednisolone pulse and subsequent oral prednisone therapy, the headache was dramatically resolved sufficiently to alleviate the need to use analgesics.

**Table 1:** Laboratory data on admission

**Peripehral blood**			**Biochemistry**					
WBC	1770	/μL	TP	6.3	g/dL	CRP	0.63	mg/dL
Neut.	49.7	%	Alb	3.8	g/dL	ESR(1h)	57	mm
Lymph.	36.7	%	T-Bil	0.6	mg/dL	ESR(2h)	102	mm
Mono.	10.2	%	CK	22	U/L	Ferritin	700	ng/ml
Eosino.	2.8	%	AST	14	U/L			
Baso.	0.6	%	ALT	30	U/L	IgG	1143	mg/dL
Blast	0.0	%	LDH	348	U/L	IgA	90	mg/dL
RBC	181	x104/μL	ALP	162	U/L	IgM	93	mg/dL
Hb	6.6	g/dL	γGTP	36	U/L	IgG4	20	mg/dL
MCV	109.4	fL	ChE	210	U/L			
MCH	36.5	pg	BUN	14.0	mg/dL	PR3-ANCA	<0.5	IU/mL
MCHC	33.3	g/dL	CRE	0.86	mg/dL	MPO-ANCA	<1.0	IU/mL
Plt	11.5	x104/μL	UA	4.3	mg/dL			
			Glu	107	mg/dL	**Coagulation**		
**Urinalysis**			Na	138	mEq/L	PT	>100	%
Protein		(−)	K	4.3	mEq/L	APTT	31.9	sec
Ocult blood		(−)	Cl	103	mEq/L	Fiblinogen	350	mg/dL
sugar		(−)	Ca	8.3	mg/dL	D-dimer	0.7	μg/mL

**Table 2:** Myelogram at diagnosis

Myeloblasts	4.5	%
Promyelocytes	0.5	%
Myelocytes	6.0	%
Metamyelocytes	1.5	%
Neutrophils	19.0	%
Eosinophil & precursors	2.5	%
Basophil & precursors	0.5	%
**Myeloid lineage**	**34.5**	％
Erythroblasts	0.0	%
Erythroid elements	13.5	%
**Erythroid lineage**	**13.5**	％
Monocytes	8.0	%
Lymphocytes	41.0	%
Plasma cells	2.0	%
Mast cells	1.0	%
**Others**	**52.0**	％
Nuclear cell count	13000/μL	
Megakaryocyte	94/μL	
Myeloid:Erythroid Ratio	2.56

**Table 3:** Chromosome analysis using G-banding method

46, XY, −3, der(5; 19)(p10; q10), −7, −9, −13, +19, 4mar	［13］
46, XY,	［7］

**Fig. 2:**
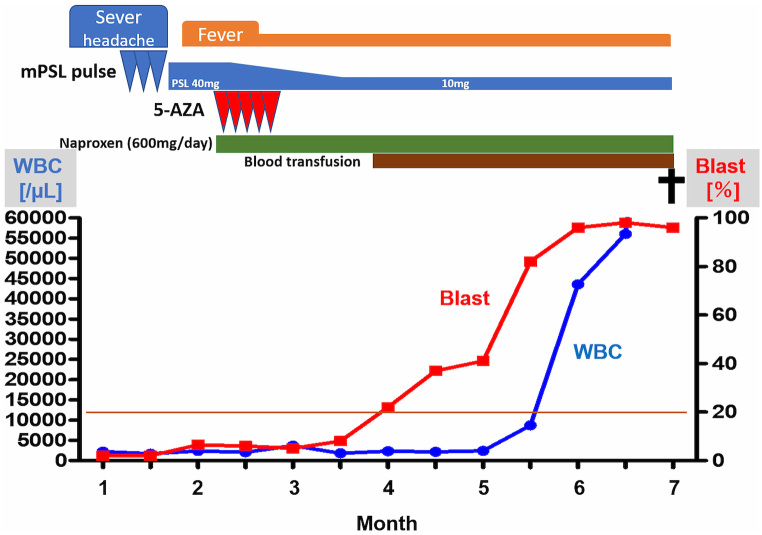
Clinical course: azacitidine (5-AZA), peripheral blood blast cells (Blast), prednisone (PSL), methylprednisolone (mPSL) and white blood cells (WBC).

After improvement of HP and just before azacitidine introduction, the fever with 38–39° in Celsius developed in the patients for weeks. Extensive examinations, including enhanced computed tomography and FDG-PET, did not identify a possible source of the fever. Repeated blood culture tests showed no positive sign of bacteremia. As a treatment for febrile neutropenia, empirical antibiotic therapy was introduced, but the fever did not resolve. These findings led to the conclusion that the fever was MDS related. Administration of an antipyretic agent had a modest fever-lowering effect. Azacitidine monotherapy was introduced; however, because of severe neutropenia with grade 4 and subsequent pneumonia, the second cycle was postponed. Two months after of azacitidine introduction, peripheral blood blast cells gradually increased. Bone marrow examination revealed 45.6 % blast cells. Despite therapy with azacitidine, MDS progressed to AML. Because of AML progression, the anemia worsened to a transfusion-dependent state. Although a low-dose cytarabine-containing regimen was considered, the patient decided to stay home without undergoing aggressive chemotherapy. Unfortunately, he died of sepsis secondary to severe neutropenia 6 months later. Throughout the clinical course of the patient's MDS and subsequent AML, there was no HP recurrence during treatment with a minimum of 10 mg prednisone (Fig. 2).

## 3 Discussion

MDS is a heterogenous group of myeloid neoplasms, not a lymphoproliferative disease; however, clinical and laboratory studies have implicated immune system derangement in the pathogenesis and progression of MDS. Actually, one-third of patients with MDS develop concomitant autoimmune disease [5]. In addition, immunosuppressive treatments such as antithymocyte globulin and cyclosporine elicit a hematological response in a fraction of patients. In a large database analysis of 2471 MDS patients from Medicare reports, the most frequent autoimmune diseases were chronic rheumatic heart disease (173 cases) and rheumatic arthritis (150 cases) [6]. In some cases of rheumatic disease complicated with MDS, immunosuppressive treatment including steroid therapy provided sufficient relief of rheumatic phenomenon but did not ameliorate the hematological condition [7], similar to the outcomes in our case. These findings suggest that the autoimmune aspect of MDS is partly mediated by cytokines and/or autoantibodies as a paraneoplastic syndrome. Autoimmunity mechanisms, mediated by activated T cells and the cytokines they trigger the release of such as TNF-α and IFN-γ, are associated with pathophysiology in some cases of MDS [8]. With HP, both T helper 1 (Th1) cytokines such as interferon (IFN)-γ and T helper 2 (Th2) cytokines such as IL-4, IL-10, and IL-13 have been implicated in dura matter fibrosis [9]. Yokoseki also reported increased levels of IL-6, C-X-C motif ligand (CXCL)-8, and CXCL-10/IFN-γ inducible protein (IP)-10 in cerebrospinal fluid of HP with ANCA-associated vasculitis or IgG4-RD [10]. It is hypothesized that inflammatory cytokines such as TNF-α and IFN-γ produced by a deranged immune system in MDS evoke an inflammatory response in the dura mater via cerebrospinal fluid invasion.

Although genetic mutations involved in the pathogenesis of MDS have been elucidated [11], a biological link between MDS and autoimmune disease has not been fully revealed. As a clinical condition linking MDS with inflammation, a new disease entity known as VEXAS syndrome has surfaced [12]. This syndrome is genetically characterized by somatic mutation affecting methionine-41 (p.Met41) in *UBA1*, an X-chromosome gene encoding ubiquitin-like modifier-activating enzyme1, and clinically with inflammatory syndrome and cytopenia, as well as recurrent fevers, pulmonary involvement, neutrophilic dermatoses, cutaneous vasculitis, macrocytic anemia, hematopoietic dysplasia, and bone marrow vacuolization restricted to myeloid and erythroid precursor cells [12].

Regrettably, our patient died before he could be examined for somatic mutations associated with VEXAS syndrome. It was uncertain whether HP was VEXAS-related condition or not. It is interesting to note that cytokine profiling in VEXAS syndrome showed increased levels of IFN-γ, CXCL-10/IP-10, and IL-8 as well as in HP [12]. A high percentage of VEXAS syndrome cases have comorbid relapsing polychondritis with inflammatory induced connective tissue disorder etiologically similar to HP [12]. These findings suggest the existence of genetic abnormalities associated with the onset of HP and MDS. Immune abnormalities associated with MDS should be genetically elucidated with further research by accumulating cases.

In conclusion, we report the case of HP accompanied with MDS. Our case provides a thought-provoking hypothesis regarding the potential relationship between clonal myeloid neoplasms and HP.

## Ethics statement

Ethical review was not required for the study on human participants in accordance with the local legislation. The informed consent was obtained from family members of the deceased patient for publication.

## Data availability statement

The authors confirm that the data supporting the findings of this study are available within the article.

## Funding

This study was supported by grants from 10.13039/501100001691Japan Society for the Promotion of Science (10.13039/501100001691JSPS) Grant-in-Aid for Scientific Research (C) (21K07237).

## CRediT authorship contribution statement

**Shohei Kikuchi:** Writing – review & editing, Writing – original draft, Project administration, Methodology, Data curation, Conceptualization. **Tomohiro Hayashi:** Data curation. **Honoka Nitta:** Data curation. **Yusuke Kamihara:** Data curation. **Akinori Wada:** Data curation. **Tomoki Minemura:** Data curation. **Yoshimi Nabe:** Data curation. **Jun Murakami:** Data curation. **Yuji Nakatsuji:** Supervision, Data curation. **Tsutomu Sato:** Supervision, Funding acquisition, Data curation.

## Declaration of competing interest

The authors declare the following financial interests/personal relationships which may be considered as potential competing interests: Tsutomu Sato reports financial support, article publishing charges, and writing assistance were provided by 10.13039/501100001691Japan Society for the Promotion of Science.
